# A Novel and Sensitive Fluorescent Probe for Glyphosate Detection Based on Cu^2+^ Modulated Polydihydroxyphenylalanine Nanoparticles

**DOI:** 10.3390/bios13050510

**Published:** 2023-04-28

**Authors:** Xiqiong Mu, Jian Xu, Fankui Zeng

**Affiliations:** 1 Research & Development Center for Eco-Material and Eco-Chemistry, Lanzhou Institute of Chemical Physics, Chinese Academy of Sciences, Lanzhou 730000, China; 2 College of Pharmacy, Gansu University of Chinese Medicine, Lanzhou 730101, China

**Keywords:** pesticide residues, fluorescent probe, polydihydroxyphenylalanine nanoparticles, Cu^2+^ ion, static quenching, glyphosate

## Abstract

A novel and sensitive fluorescent probe based on Cu^2+^-modulated polydihydroxyphenylalanine nanoparticles (PDOAs) has been developed for the detection of glyphosate pesticides. Compared to conventional instrumental analysis techniques, fluorometric methods have obtained good results in the field of agricultural residue detection. However, most of the fluorescent chemosensors reported still have some limitations, such as long response times, the high limit of detection, and complex synthetic procedures. In this paper, a novel and sensitive fluorescent probe based on Cu^2+^ modulated polydihydroxyphenylalanine nanoparticles (PDOAs) has been developed for the detection of glyphosate pesticides. The fluorescence of PDOAs can be effectively quenched by Cu^2+^ through the dynamic quenching process, which was confirmed by the time-resolved fluorescence lifetime analysis. In the presence of glyphosate, the fluorescence of the PDOAs-Cu^2+^ system can be effectively recovered due to the higher affinity of glyphosate for Cu^2+^, and thus released the individual PDOAs. Due to the admirable properties such as high selectivity to glyphosate pesticide, “turn on” fluorescence response, and ultralow detection limit of 1.8 nM, the proposed method has been successfully applied for the determination of glyphosate in environmental water samples.

## 1. Introduction

Recently, pesticide residue and food safety issues have always been a topic of concern and present a significant challenge all over the world [1,2,3]. Although the extensive use of pesticides and herbicides can effectively improve the yield of agricultural crops, unreasonable application of pesticides is still a major threat to human health and public safety. Glyphosate (*N*-[phosphine methyl] glycine), as a kind of broad-spectrum and non-selective herbicide, has been widely used worldwide for its excellent performance and effectiveness in weed control [4,5]. However, the abuse of glyphosate often results in high residues in environmental samples because of its high water solubility, long half-life, and high retention in soil, which may pose a risk to human health [6,7,8]. For example, glyphosate can irreversibly inactivate acetylcholinesterase (AChE), resulting in respiratory, myocardial, and neuromuscular dysfunction and even death [9,10,11]. In addition, recent studies have shown that glyphosate has potential carcinogenic effects in humans and inhibits the production of male steroid hormones, which may lead to reduced fertility [12]. As a result, the World Health Organization (WHO) has set a maximum contaminant level of glyphosate residue in drinking water at 5.325 μM (900 μg/L) [13].

In the past decade, many efforts have been devoted to the development of detection techniques for pesticides, including liquid/gas chromatography [14,15], mass spectrometry [16], immunoassay [17], surface-enhanced Raman scattering [18] and electrochemistry [19]. Although these conventional methods displayed high selectivity and adequate sensitivity for pesticide detection, they still involve some limitations for the need for tedious sample pretreatment, qualified analytical technicians, and expensive equipment, which greatly limits their application in actual sample analysis and detection. Compared to these instrumental analysis systems, colorimetric or fluorometric methods have attracted more research interest for their rapid analysis speed, simple operation procedures, high detection sensitivity, and simple instruments [20,21,22]. However, due to the lack of molecular recognition sites in the molecular structure of glyphosate, it is still a challenge to design and develop ideal chromophores and fluorescent detection systems for glyphosate. To date, only a few excellent optical sensors have been developed for pesticide residue sensing. For example, Jiang and co-workers reported a dual-readout assay based on gold nanoparticles for the detection of organophosphorus and carbamate pesticides [23]. Mang and co-workers constructed a simple fluorescent sensor for methyl parathion detection based on an L-tyrosine functionalized carbon dots (Tyr-CDs) system [24]. Despite the fine effects of pesticide detection, these reported chemosensors still have some limitations, such as long response times, the high limit of detection, and complex synthetic procedures. Therefore, there is an increasing demand for establishing a reliable method for the rapid, simple, and sensitive determination of glyphosate in environmental samples.

Recently, it is reported that polydopamine (PDA) organic nanoparticles can be effectively synthesized through the self-polymerize of Dopamine (DA) under alkaline conditions [25]. PDA nanoparticles have been applied in various fields including catalysis, metal deposition, surface modification, cancer diagnosis, drug delivery, and biochemical sensing [26,27,28]. Bayindir and co-workers developed a facile assay for the synthesis of polydopamine nanoparticles with intensive green fluorescence emission [25]. Wu and co-workers prepared highly emissive and biocompatible dopamine-derived oligomers for Fe^3+^ ion sensing and targeted bioimaging [29]. Zhang and co-workers reported an effective fluorescent Cu^2+^ detection method using PDA nanoparticles [30]. However, to the best of our knowledge, the reported PDA-based fluorescent sensors for pesticide or glyphosate detection are still rare.

**Scheme 1:**
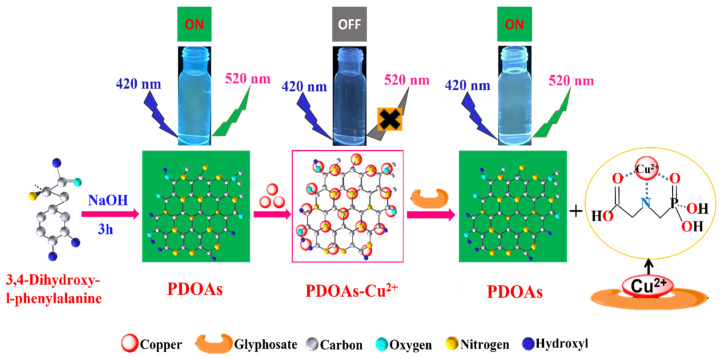
The mechanism of glyphosate detection by PDOAs-Cu^2+^ complex.

In this work, a novel fluorescent probe based on Cu^2+^ modulated polydihydroxyphenylalanine nanoparticles (PDOAs) has been developed for the detection of glyphosate pesticide residues for the first time. Dihydroxyphenylalanine (DOA) is the precursor of dopamine in biological processes [31], which has a similar structure to dopamine (DA). PDOAs were prepared by a simple synthetic process under alkaline conditions. As shown in Scheme 1, the PDOAs displayed obvious green fluorescence. Interestingly, the fluorescence of PDOAs can be selectively quenched by Cu^2+^ and the sensing mechanism is ascribed to the multi-coordination interaction between Cu^2+^ with N atoms, O atoms, amino, carboxyl, and catechol groups in the molecule structure of PDOAs. According to previous reports, glyphosate can strongly chelate with Cu^2+^ and the binding affinity is higher than other metal ions [32,33,34]. Inspired by these findings, we speculate that the PDOAs-Cu^2+^ complex system can be utilized for glyphosate detection. As expected, the addition of glyphosate could quickly restore the fluorescence of the PDOAs-Cu^2+^ system because of the competitive displacement of glyphosate for PDOAs, which is originated from the strong coordination of carboxyl (-COOH), phosphonyl (-PO(OH)_2_), and imine (-NH-) of glyphosate with Cu^2+^. Subsequently, the free PDOAs were released, which finally resulted in the recovery of green emission and color change from colorless to pale yellow. Based on this strategy, the well-designed fluorescent probe based on the PDOAs-Cu^2+^ complex was applied to the sensitive and selective detection of glyphosate with a low detection limit of 0.799 nM. In addition, the mechanism investigation indicated that the fluorescence quenching of PDOAs by Cu^2+^ was ascribed to the static quenching process, and the corresponding fluorescence lifetimes of the sensing system were measured. In view of the favorable property, the novel method has been successfully applied for the detection of glyphosate in environmental water samples.

## 2. Materials and Methods

### 2.1. Starting Materials and Instruments

Sodium hydroxide, CuSO_4_, 3,4-Dihydroxy-phenylalanine, and other metal ions were purchased from Sigma-Aldrich reagent company (Shanghai, China). *N*-2-hydroxyethylpiperazine-*N*-ethane-sulphonic acid (HEPES) was purchased from Aladdin chemistry company (Shanghai, China). All reagents were of analytical grade and used without further purification. Water was purified by a Milli-Q system. UV–vis absorbance spectrum measurements were performed on a PerkinElmer Lambda 750S UV–visible Spectrophotometer (PerkinElmer, Waltham, MA, USA). Fluorescent spectrum and lifetime were measured on a HORIBA Spectro fluorophotometer (Horiba, Kyoto, Japan). X-ray photoelectron spectroscopy (XPS) was conducted on a Thermo Fisher Scientific Escalab 250Xi photoelectron spectrometer (Thermo Fisher, Waltham, MA, USA). The scanning electron microscope images were captured on a SU8020 ultra-high resolution field emission scanning electron microscope (Hitachi, Tokyo, Japan).

### 2.2. Preparation of PDOA Nanoparticles

The preparation of PDOA nanoparticles were accorded to the reported method with minor change [25]. In short, 19.9 mL dihydroxyphenylalanine solution (1 mmol/L) was mixed with 0.1 mL NaOH solution (1 mol/L). The above solution was stirred for 3 h at room temperature (25 °C). Then, an HCl solution (1 mol/L) was applied to terminate the polymerization reaction. The pH of the final solution was adjusted to about 7.0. Subsequently, the solution was dialyzed for 24 h to obtain the purified PDOA nanoparticles. After dialysis, the as-prepared PDOAs solution (20 mL) was concentrated and freeze-dried to afford the desired PDOAs as a solid powder (0.068 g). Thus, the concentration of the PDOAs matrix solution was determined to be 3.4 mg/mL (Supporting Information). In the following experiments, the PDOAs solution was diluted 10 times to 0.34 mg/mL for all the spectrometric analysis and glyphosate detection.

### 2.3. Fluorescence Detection of Cu^2+^ and Glyphosate

The spectroscopic experiments were carried out in HEPES buffer solution (pH 7.5) as follows. In a series of colorimetric tubes, the PDOAs stock solution (400 μL, 3.4 mg/mL) was mixed with Cu^2+^ standard solution. The Cu^2+^ concentrations were 0, 0.4, 0.8, 1.2, 1.6, 2.0, 2.4, 2.8, 3.2, 3.6, 4.0, 4.4, 4.8, and 5.2 μM, respectively. Then, the mixed solution was diluted to 4 mL with HEPES buffer solution. The final concentration of PDOAs was 0.34 mg/mL. After incubation for 15 min at room temperature, the fluorescence spectra of the measuring solutions were recorded under an excitation wavelength of 420 nm. The linear relationship between the fluorescence intensity of PDOAs and Cu^2+^ concentration was obtained. The procedure for glyphosate determination was shown as follows. A total of 400 μL of PDOAs stock solution (3.4 mg/mL) was firstly incubated with 3.6 μM Cu^2+^ at room temperature for 15 min. After that, the formed PDOAs-Cu^2+^ system was incubated with HEPES buffer solution (pH 7.5) containing different concentrations of glyphosate (0, 0.1, 0.2, 0.3, 0.4, 0.5, 0.6, 0.7, 0.8, 0.9, 1.0, 1.1, 1.2, 1.3, 1.4, 1.5, 1.7, 2.0, 2.5 and 3.0 μM). The resulting mixture solution was diluted to 4 mL with HEPES buffer solution and then incubated for 30 min. The fluorescence spectra of the measuring solutions were recorded from 460 nm to 650 nm with the excitation at 420 nm. The linear relationship between the fluorescence intensity of the PDOAs-Cu^2+^ system and the glyphosate concentration was obtained.

### 2.4. Detection of Glyphosate in Real Environmental Water Samples

In order to investigate the practical application of the detection method, real water samples were studied. The various water samples were collected from tap water and the Yellow River (Lanzhou, China). Before the fluorescent detection, the water-insoluble organics and particle impurities were filtered through 0.22 μm microporous membranes. Then the glyphosate standard solution was added to actual water samples. The final concentrations of glyphosate in the actual samples were 0.50, 0.75, 1.0, and 1.25 µM, respectively. Finally, the fluorescence intensity was measured under the optimum conditions. The concentrations of glyphosate in these water samples were calculated according to the working curve and each sample was tested three times.

## 3. Results and Discussion

### 3.1. Characterization of PDOAs

**Figure 1:**
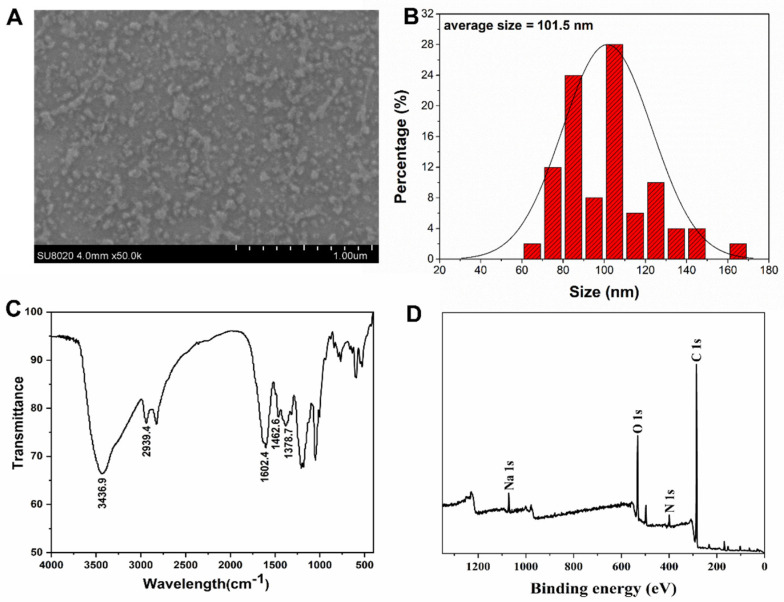
(**A**) The morphologic analysis of the PDOA nanoparticles by HR-SEM. (**B**) Size distribution of the PDOA nanoparticles. (**C**) FTIR spectrum of the PDOA nanoparticles. (**D**) XPS spectrum analysis of the PDOA nanoparticles.

Under alkaline conditions, dihydroxyphenylalanine (DOA) will be oxidized to the quinone derivative and then autopolymerized to form PDOA nanoparticles (PDOAs). Through the high-resolution scanning electron microscope (HR-SEM) analysis, the morphology of the obtained PDOA nanoparticles can be investigated in depth. As displayed in Figure 1A, PDOAs were irregular in shape and showed a wide particle size distribution. The size of PDOAs was mainly distributed in the range of 80–110 nm, with an average size of 101.5 nm (Figure 1B).

To confirm the functional groups on the surface of PDOAs, the Fourier transform infrared spectrometer (FTIR) analysis was performed. As shown in Figure 1C, the intense peak at 3436.9 cm^−1^ was ascribed to the vibration of the -OH group. The peaks centered at 1602.4 cm^−1^ and 1462.6 cm^−1^ were attributed to the asymmetric and symmetric stretching vibrations of the COO^-^ group, respectively. The band at 1378.7 cm^−1^ was due to the C-N stretching vibrations. The above results indicated the presence of hydroxy, carboxyl, and imine groups. These functional groups not only improve the hydrophilicity of the PDOAs for sensing applications, but may also act as Cu^2+^ binding sites.

Next, X-ray photoelectron spectroscopy (XPS) analysis was carried out. Four typical peaks of C_1s_, O_1s_, N_1s_, and Na_1s_ were shown in the XPS survey spectrum (Figure 1D). Through semi-quantitative analysis of XPS, the atomic percentages of C, O, N, and Na of the sample were measured to be 78.38%, 15.58%, 4.24%, and 1.79%, respectively. The detailed results of C_1s_, O_1s_, and N_1s_ spectra are displayed in Figures S1–S3. The C_1s_ spectrum shows the existence of C–C, C–N, C–O and C=O groups, which are in accordance with the FTIR data (Figure S1). The high-resolution O_1s_ spectrum consisted of two peaks with the binding energy of 531.8 eV and 533.5 eV, which were assigned to the C–O and O=C–O groups (Figure S2). The N_1s_ spectrum (Figure S3) displayed three nitrogen states including pyridine nitrogen (N–C, 399.2 eV), pyrrolic nitrogen (N–C=C, 401.6 eV), and graphitic nitrogen (N–H, 401.6 eV).

### 3.2. Spectroscopic Property of PDOAs

**Figure 2:**
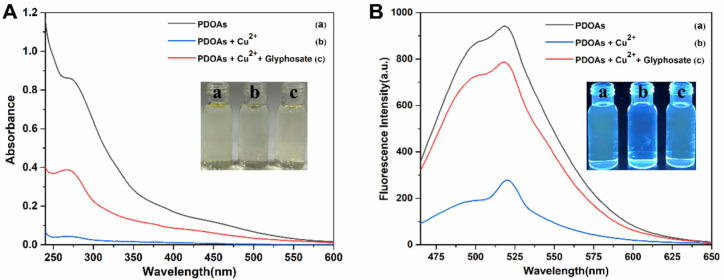
Absorption (**A**) and fluorescence (**B**) spectra: (a) 0.34 mg/mL PDOAs; (b) 0.34 mg/mL PDOAs in the presence of 3.6 μM Cu^2+^; (c) the reaction mixture of (b) with 1.5 μM glyphosate in HEPES buffer solution (pH 7.5). The inset displays the color picture of (**A**) and the fluorescent picture of (**B**).

Initially, spectroscopic evaluation of PDOAs was carried out in HEPES buffer solution (pH 7.5). Individual PDOAs exhibit a maximum absorbance of 270 nm. Upon addition of Cu^2+^ to the PDOAs solution, the absorption peak at 270 nm decreased and gradually disappeared (Figure 2A), which was reasonably ascribed to the coordination interaction between PDOAs and Cu^2+^. Correspondingly, the color of the solution changed prominently from faint yellow to colorless. When adding glyphosate to PDOAs-Cu^2+^ complex system, the color of the solution is recovered and changed from colorless to faint yellow.

In the fluorescent channel, PDOAs showed a characteristic fluorescence emission peaks at 520 nm, when excited at 420 nm. After adding Cu^2+^ to the PDOAs solution, the fluorescence emission peak at 520nm was significantly decreased. Accordingly, the bright green-colored fluorescence obviously disappeared (Figure 2B). Upon adding glyphosate, the fluorescence “off” state of the PNDPs-Cu^2+^ system is resumed due to the strong chelate action of glyphosate to Cu^2+^. Subsequently, the free PDOA nanoparticles were released, which finally resulted in the recovery of green emission (fluorescence “on” state) and color change from colorless to pale yellow.

### 3.3. Cu^2+^ Detection Based on “Turn Off” Fluorescence of PDOAs

**Figure 3:**
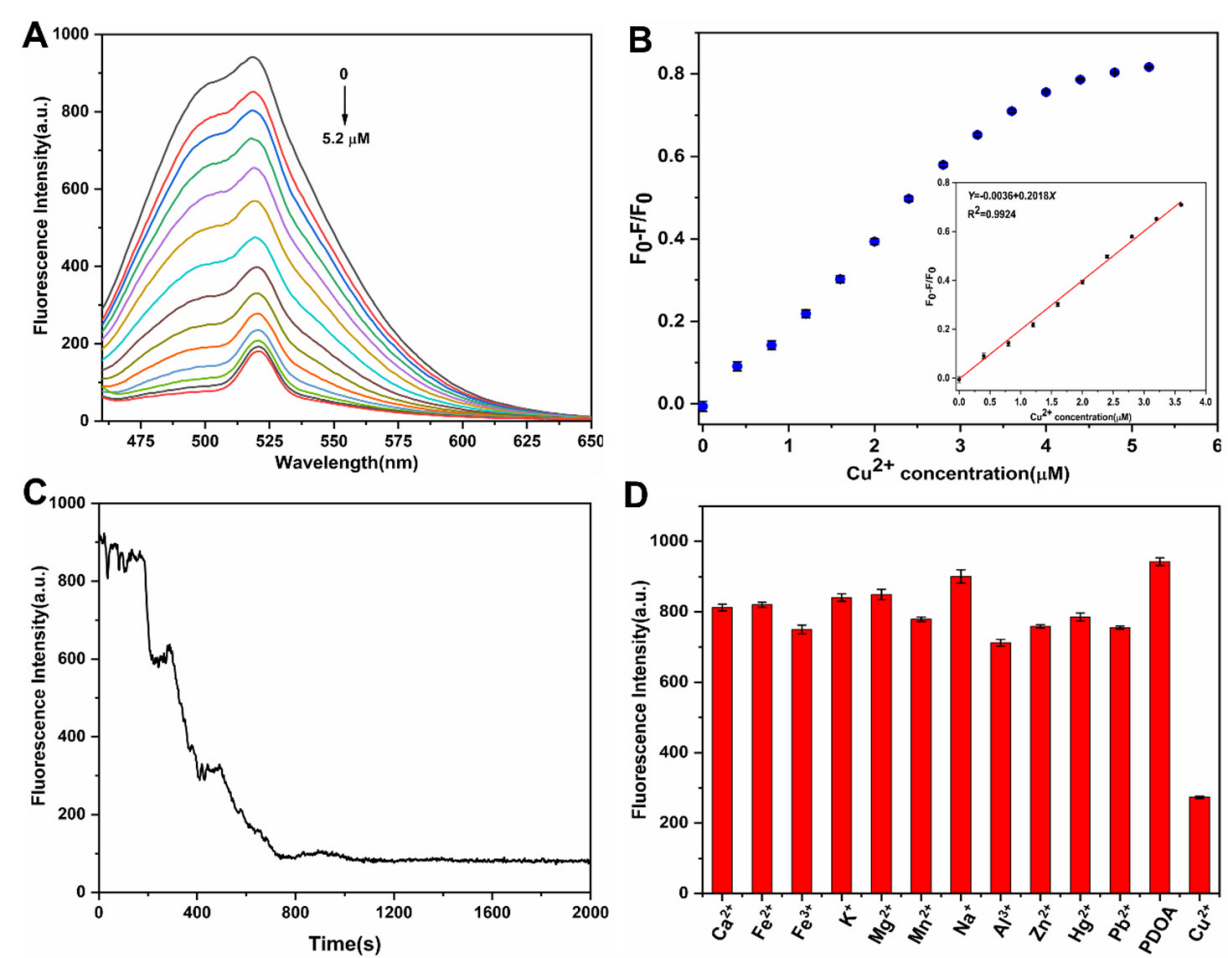
(**A**) Fluorescence response of PDOAs (0.34 mg/mL) to different concentrations of Cu^2+^ (0, 0.4, 0.8, 1.2, 1.6, 2.0, 2.4, 2.8, 3.2, 3.6, 4.0, 4.4, 4.8 and 5.2 μM). (**B**) The corresponding linear range of PDOAs after adding various concentrations of Cu^2+^. (**C**) Time response of 0.34 mg/mL PDOAs to 3.6 μM Cu^2+^. (**D**) Fluorescent responses of 0.34 mg/mL PDOAs toward 10 μM various metal cations. Every data point was the mean of three measurements. The error bars are the standard deviation.

We then moved forward to the emission titration experiments. (Figure 3A). With the addition of various concentrations of Cu^2+^, the fluorescence intensity of the PDOAs solution was gradually decreased. The ratio of fluorescent intensity at 520 nm (F_0_-F/F_0_) displayed a good linear response with the concentration of Cu^2+^ in the range of 0–3.6 μM (Figure 3B). In addition, the LOD value was measured to be 1.69 nM (3 *б*/*k*, in which *б* is the standard deviation of blank measurements, and *k* is the slope of the linear equation). It is noted that about 71.14% reduction in the fluorescence intensity is obtained upon the addition of 3.6 μM of Cu^2+^, which makes the PDOAs-Cu^2+^ complex system a good platform for the next step detection of glyphosate.

To further optimize the detection conditions, the time response of PDOAs to Cu^2+^ was further investigated. As shown in Figure 3C, after adding Cu^2+^ to the sensing system, a prompt fluorescent decrease at 520 nm was observed and the reaction equilibrium was almost approached at about 15 min.

Furthermore, the selectivity of the sensing system was evaluated by treatment of various potential interfering species, such as usually used cations (Ca^2+^, Fe^2+^, Fe^3+^, K^+^, Mg^2+^, Mn^2+^, Na^+^, Al^3+^, Zn^2+^, Hg^2+^ and Pb^2+^). From Figure 3D, we can see that the fluorescence of the PDOAs solution was only significantly quenched by Cu^2+^ ion. In contrast, neglectable fluorescent intensity changes were found after the addition of other interfering cations under the same conditions.

The pH effect was further investigated for Cu^2+^ sensing (Figure S4). After adding Cu^2+^ to the PDOAs solution, the ratio of fluorescent intensity at 520 nm (F_0_-F/F_0_) slightly decreased when the pH value was below 7.0, which may be ascribed to the protonated carboxyl group (or hydroxyl, amine group) and thus inhibited the coordination of PDOAs with Cu^2+^. Therefore, the neutral and weakly alkaline conditions are favorable for the detection of Cu^2+^.

### 3.4. Glyphosate Detection Based on “Turn On” Fluorescence of PDOAs-Cu^2+^ System

**Figure 4:**
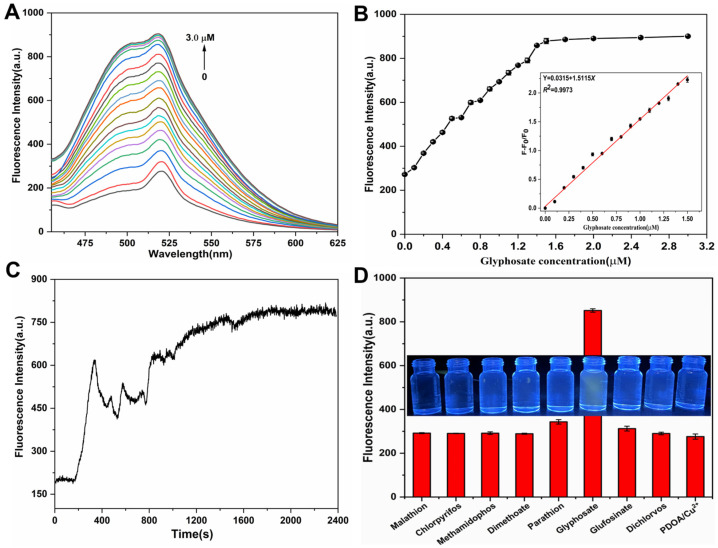
(**A**) Fluorescence spectra of the PDOAs-Cu^2+^ system in the presence of different concentrations of glyphosate (0, 0.1, 0.2, 0.3, 0.4, 0.5, 0.6, 0.7, 0.8, 0.9, 1.0, 1.1, 1.2, 1.3, 1.4, 1.5, 1.7, 2.0, 2.5 and 3.0 μM). (**B**) The corresponding linear range of PDOAs-Cu^2+^ upon addition of different concentrations of glyphosate. (**C**) Time response of the PDOAs-Cu^2+^ system toward 1.5 μM glyphosate. (**D**) Fluorescence responses of PDOAs-Cu^2+^ to 10 μM various organophosphorus pesticides.

In previous works, the strong chelate interaction of glyphosate with Cu^2+^ has been reported [32,33,34]. Therefore, we speculated that the PDOAs-Cu^2+^ system can be an effective candidate for glyphosate detection. To verify the expected principle for glyphosate detection, the interaction of glyphosate with the PDOAs-Cu^2+^ system was carefully investigated. As shown in Figure 4A, the fluorescent intensity of the PDOAs-Cu^2+^ system was gradually enhanced with increasing glyphosate concentrations in the range of 0–3.0 µM. In addition, the fluorescence intensity ratio (F-F_0_/F_0_) at 520 nm showed a good linear response with the concentration of glyphosate in the range of 0–1.5 μM (R^2^ = 0.9973), where F and F_0_ represent the fluorescence intensity of PDOAs-Cu^2+^ system in the presence and absence of glyphosate (Figure 4B). The detection limit was measured to be 1.8 nM (3 *б*/*k*, in which *б* is the standard deviation of blank measurements, and *k* is the slope of the linear equation). As shown in Table S1, the LOD of the proposed method for detecting glyphosate was almost better than the previously reported methods [35,36,37,38].

To understand the kinetic reaction mechanism, the time-dependent fluorescence responses of the PDOAs-Cu^2+^ system to glyphosate were investigated (Figure 4C). The addition of 1.5 μM glyphosate led to a dramatic increase in the fluorescence intensity of the PDOAs-Cu^2+^ system and reached equilibrium at about 25 min. In order to obtain the maximum fluorescent recovery and stable signal, 30 min was selected as the optimum incubation time for the detection of glyphosate.

For a reliable fluorescence probe, high selectivity is another significant requirement for analyte detection. In order to demonstrate the selectivity of the proposed method for target detection, the fluorescence responses of the PDOAs-Cu^2+^ system to some pesticides such as malathion, chlorpyrifos, methamidophos, dimethoate, parathion, glufosinate and dichlorvos were performed. As shown in Figure 4D, upon excitation at 420 nm, only glyphosate induced a large fluorescence enhancement at 520 nm, whereas other interfering species triggered a small or negligible fluorescence variation. The results demonstrated that the PDOAs-Cu^2+^ system displayed high selectivity for glyphosate detection.

### 3.5. Investigation of Sensing Mechanism

In order to further investigate the mechanism of fluorescence quenching of PDOAs upon the addition of Cu^2+^, the Stern-Volmer equations were used for analyzing the respective emission data (Equation (1)).

(1) $$\begin{array}{l} \frac{F_{0}}{F}=1+K_{SV}[Q]=1+K_{q}\tau_{0}[Q]\\ K_{q}=\frac{K_{SV}}{\tau_{0}} \end{array}$$

**Figure 5:**
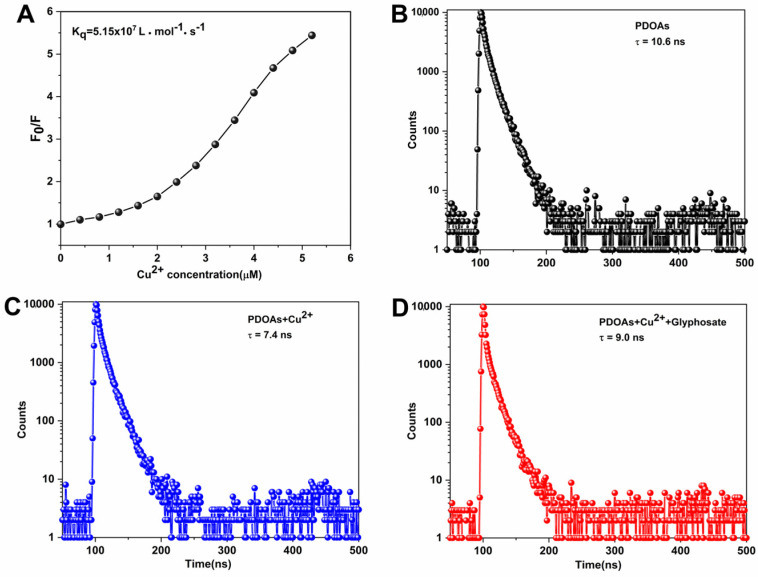
(**A**) The Stern-Volmer plots of PDOAs upon addition of Cu^2+^ with different concentrations. (**B**) Fluorescence decay curves of PDOAs and corresponding fluorescence lifetime. (**C**) Fluorescence decay curves and fluorescence lifetime of PDOAs in the presence of Cu^2+^. (**D**) Fluorescence decay curves and fluorescence lifetime of PDOAs-Cu^2+^ system in the presence of glyphosate.

In the equations, *F*_0_ and *F* represent the emission intensity of PDOAs in the absence and presence of a quencher, respectively. *K_SV_* is the Stern–Volmer quenching constant, [*Q*] is the concentration of quencher and *τ*_0_ represents the average lifetime of the PDOAs in the absence of Cu^2+^. Figure 5A showed the Stern-Volmer plots of PDOAs in the presence of various concentrations of Cu^2+^. The *K_q_* value was calculated to be 5.15 × 10^7^ L mol^−1^ s^−1^, which indicated that the probable quenching mechanism of PDOAs by Cu^2+^ ions was ascribed to the dynamic quenching rather than static quenching [39,40].

Furthermore, time-resolved fluorescence lifetime measurement was carried out to distinguish the static and dynamic quenching mechanism of PDOAs with Cu^2+^. The fluorescence lifetime measurement of PDOAs in the absence and presence of Cu^2+^ was illustrated in Figure 5B,C. The average fluorescence lifetime value of individual PDOAs is measured to be 10.6 ns. Upon the addition of 3.6 μM Cu^2+^ to the PDOAs solution, the fluorescence lifetime changed to 7.4 ns. The significant change in the average fluorescence lifetime clearly indicated that the fluorescence quenching belongs to dynamic quenching, reflecting the strong interaction between Cu^2+^ and PDOAs.

In addition, when glyphosate was added to the PDOAs-Cu^2+^ complex solution, the fluorescence lifetime of the system was increased to 9.0 ns (Figure 5D), which is close to the value of individual PDOAs. The results further demonstrated the fluorescence recovery originated from the strong coordination interaction between glyphosate and Cu^2+^, which resulted in the release of free PDOAs.

### 3.6. Detection of Glyphosate in Real Samples

To evaluate the practicability of the proposed method, a practical sample analysis was carried out to the detection of glyphosate in the environmental water samples. Laboratory tap water and Yellow River water were chosen for the spiked experiment (Table 1). The recoveries of glyphosate were investigated by adding a certain amount of glyphosate standard solution into the water samples with concentrations from 0.5 and 1.25 μM. For the spiked water samples, the recovery values were calculated to be 90.4–104.5 %, and the RSDs were no more than 5.0 %. The results indicate that the proposed method displays satisfied practicability and reliability for glyphosate detection in real water samples.

## 4. Conclusions

In summary, a novel and sensitive fluorescent probe based on Cu^2+^-modulated polydihydroxyphenylalanine nanoparticles (PDOAs) has been developed for the detection of glyphosate. Particularly, the fluorescence of PDOAs can be effectively quenched by Cu^2+^ through the static quenching process, which was confirmed by the time-resolved fluorescence lifetime analysis. When glyphosate was added to the PDOAs-Cu^2+^ system, the fluorescent signal was effectively recovered, which was ascribed to the higher affinity of glyphosate for Cu^2+^ and thus released the individual PDOAs. Based on the fluorescence quenching and recovery phenomenon, a new type of PDOAs-based fluorescence probe has been developed for the detection of Cu^2+^ with “on-off” mode and glyphosate detection with “off-on” mode. In addition, the PDOAs-based fluorescence probe has been successfully applied for the detection of glyphosate in environmental water samples with satisfactory results, indicating that this novel method is hopeful to serve as a practical tool for pesticides-related applied research and inspire the production of new lab-on-chip sensor devices.

## Figures and Tables

**Table 1:** Recovery experiments of glyphosate detection in real water samples.

Sample	Added (uM)	Found (uM)	Recovery (%)	RSD (%)
**Laboratory tap water**	0.5	0.46	92.7	1.32
	0.75	0.68	90.4	0.71
	1	0.92	92.2	1.54
	1.25	1.13	91.6	0.87
**Yellow River**	0.5	0.52	104.5	1.67
	0.75	0.74	98.5	1.19
	1	1.02	102.2	1.13
	1.25	1.28	102.6	0.43

## Data Availability

Experimental data associated with this research are available from the authors.

## Supplementary Materials

The following supporting information can be downloaded at: https://www.mdpi.com/article/10.3390/bios13050510/s1 [41,42], Figure S1: The high-resolution C1s spectrum of PDOAs; Figure S2: The high-resolution O1s spectrum of PDOAs; Figure S3: The high-resolution N1s spectrum of PDOAs; Figure S4: The fluorescence intensity ratio histogram of 0.34 mg/mL PDOAs in the presence of 3.6 μM Cu^2+^ with different pH values; Figure S5: The fluorescence intensity ratio histogram of PDOAs-Cu^2+^ system in the presence of 1.5 μM glyphosate with different pH values; Table S1: Comparison of various glyphosate probes [9,23,32,33,34,43,44,45,46]; Table S2: Fitting parameters for time-resolved fluorescence decay assay.
