# Supplementary material for: A Novel and Sensitive Fluorescent Probe for Glyphosate Detection Based on Cu2+ Modulated Polydihydroxyphenylalanine Nanoparticles
Source: Biosensors (Basel). 2023 Apr 28;13(5):510. doi: 10.3390/bios13050510 (PMC10216732; doi:10.3390/bios13050510)
Supplement: Supplementary file 1 [file biosensors-13-00510-s001.zip › biosensors-2337231-supplementary.pdf]

biosensors MDPI

Supplementary Materials

A novel and sensitive fluorescent probe for glyphosate detection based on Cu^{2+} modulated polydihydroxyphenylalanine nanoparticles

Xiqiong Mu,[a,b] Jian Xu [a,*] and Fankui Zeng [a,*]

[a] Research & Development Center for Eco-material and Eco-Chemistry, Lanzhou Institute of Chemical Physics, Chinese Academy of Sciences, Lanzhou 730000, P. R. China.

[b] College of Pharmacy, Gansu University of Chinese Medicine, Lanzhou 730101, P. R. China.

[*]Corresponding author, E-mail: xujian1980@licp.cas.cn; zengfk@licp.cas.cn

Concentration Determination of PDOA nanoparticles

After dialysis, the as-prepared PDOAs solution (20 mL) was concentrated and freeze-dried to afford the desired PDOAs as a solid powder (0.068 g). Thus, the concentration of PDOAs matrix solution was determinate to be 3.4 mg/mL.[1-2] In the following experiments, the PDOAs solution was diluted 10 times to 0.34 mg/mL for all the spectrometric analysis and glyphosate detection.

XPS Spectrum of the PDOAs

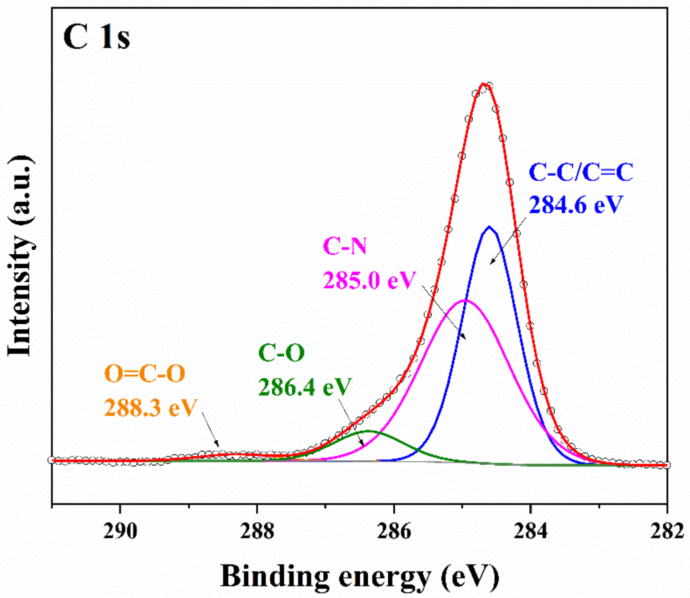

Figure S1. The high-resolution C1s spectrum of PDOAs.

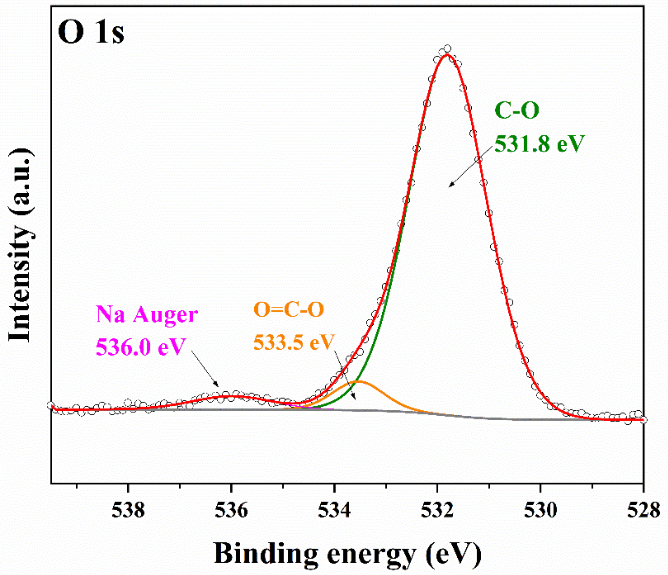

Figure S2. The high-resolution O1s spectrum of PDOAs.

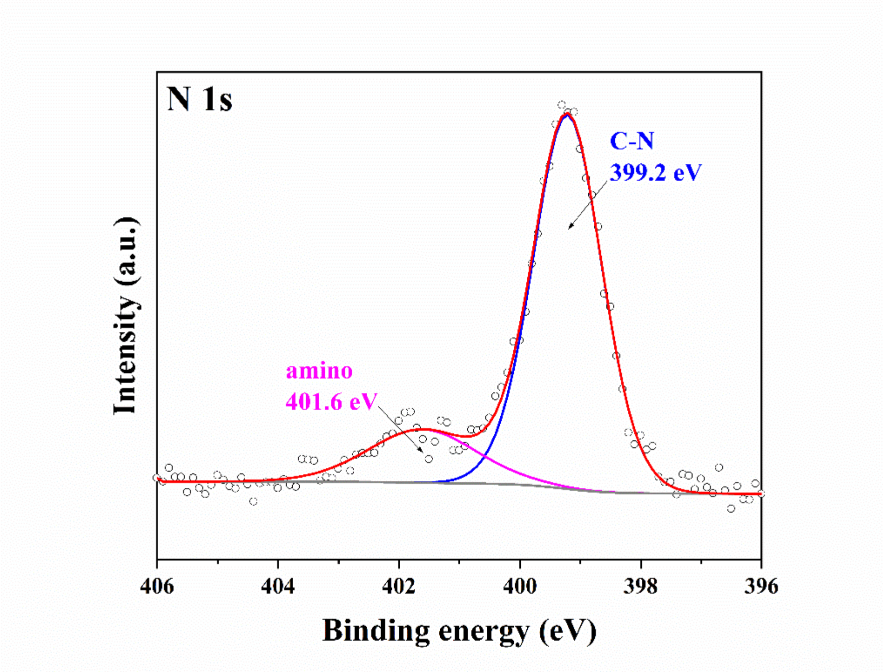

Figure S3. The high-resolution N1s spectrum of PDOAs.

Effects of pH

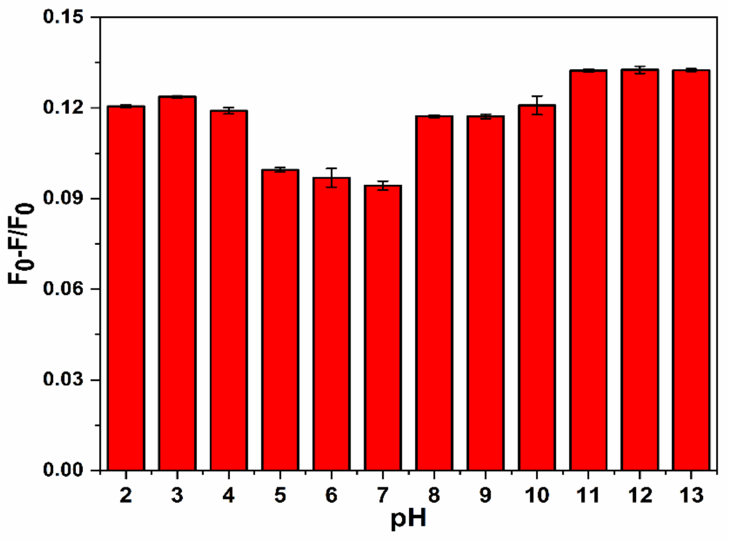

Figure S4. The fluorescence intensity ratio histogram of 0.34 mg/mL PDOAs in the presence of 3.6 μM Cu^{2+} with different pH values.

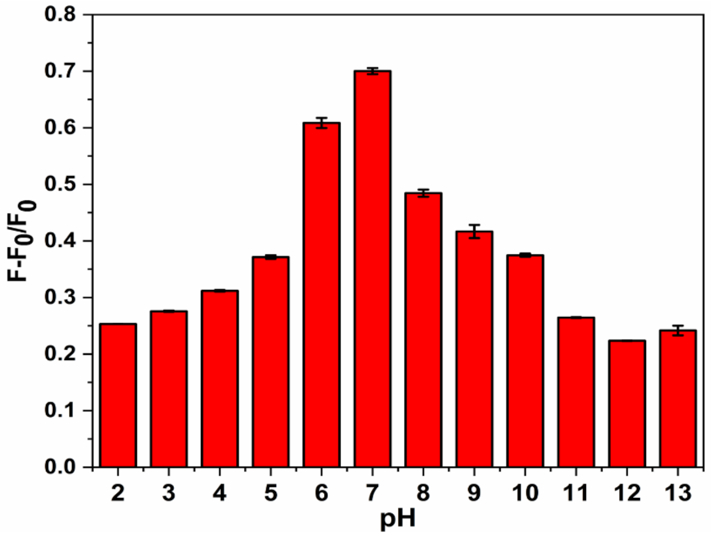

Figure S5. The fluorescence intensity ratio histogram of PDOAs-Cu^{2+} system in the presence of 1.5 μM glyphosate with different pH values.

Table S1

Comparison of various glyphosate probes.

Method/ probe	Response time (min)	Detection limit (μM)	Reference
Coumarin derivative/Cu^{2+}	5	0.11	*Anal. Methods,* 2020, 12, 520.
AuNPs/Pb^{2+}	15	2.4×10^{-3}	*Anal. Methods,* 2017, 9, 2890.
DNA-AgNCs/Cu^{2+}	1	3×10^{-2}	*Food Chem,* 2022, 367, 130617.
Rhodamine B/AuNPs	5	5.9×10^{-4}	*Anal. Chem.,* 2012, 84, 4185.
AuNPs/Cys	15	5.9	*Analyst,* 2019, 144, 2017.
Rhodamine/Cu^{2+}	2	4.1×10^{-3}	*Talanta,* 2021, 224, 121834.
CDs/Cu^{2+}	12	0.095	*RSC Adv,* 2016, 6, 85820.
lgG-CDs	120	0.047	*J. Agric. Food. Chem.,* 2016, 64, 6042.
GMP/Tb@GMP/Eu/DPA	30	41	*Food Chem,* 2020, 323, 126815.
PDOAs/Cu^{2+}	30	1.8×10^{-3}	this work

Table S2

Fitting parameters for time-resolved fluorescence decay assay.

Sample	α_1	T_1	α_2	T_2	τ(ns)	Fit	$\bar{\tau}$ (ns)
	8606.5325	5.0349	2047.2495	17.0621	10.4029	0.9965	
**PDOAs**	8869.6384	5.3429	1907.7200	17.6871	10.4768	0.9957	10.5804
	8778.6384	5.2429	1978.7200	18.0871	10.8615	0.9904	
	10292.8837	3.3920	773.0701	21.1665	7.2390	0.9962	
**PDOAs+Cu^{2+}**	1915.1589	14.0577	8354.2452	2.5173	7.4383	0.9957	7.4040
	1872.3141	13.9476	8390.9496	2.4453	7.5347	0.9952	
	8476.1712	4.6468	2197.1833	16.3567	9.0641	0.9832	
**PDOAs+Cu^{2+}+ Glyphosate**	2105.8856	12.0988	8639.4700	3.4230	8.9966	0.9977	8.9824
	8842.6799	4.9726	1965.1560	17.6361	8.8866	0.9976	

T-Fluorescence lifetime component; **α-**The amplitude of the fluorescence lifetime component; **Fit-**Fitting parameters; **τ-**mean fluorescence lifetime.

Reference

(1) Qu, Z.; Na, W.; Nie, Y.; Su, X. *Anal. Chim. Acta.* **2018**, *1039*, 74-81.

(2) Zhang, L.; Zhang, X.; Hu, B.; Shen, L.; Chen, X.; Wang, J. *Analyst* **2012**, *137*, 4974-4980.
